# Right To Play’s intervention to reduce peer violence among children in public schools in Pakistan: a cluster-randomized controlled trial

**DOI:** 10.1080/16549716.2020.1836604

**Published:** 2020-11-03

**Authors:** Rozina Karmaliani, Judith McFarlane, Hussain Maqbool Ahmed Khuwaja, Yasmeen Somani, Shireen Shehzad Bhamani, Tazeen Saeed Ali, Nargis Asad, Esnat D. Chirwa, Rachel Jewkes

**Affiliations:** a School of Nursing and Midwifery, Aga Khan University, Karachi City, Pakistan; b College of Nursing, Texas Woman’s University, Denton, TX, USA; c Department of Psychiatry, Aga Khan University, Karachi City, Pakistan; d Gender & Health Research Unit, South Africa Medical Research Council, Pretoria, South Africa; e School of Public Health, University of Witwatersrand, Johannesburg, South Africa

**Keywords:** Adolescent mental health, peer violence perpetration, peer violence victimization, school violence, empowerment, Asia, corporal punishment, gender attitudes

## Abstract

**Background:**

Peer violence is common globally, but a little researched topic in low-and middle-income countries. This study presents the evaluation of a two-year randomized controlled trial of a structured play-based life-skills intervention implemented in schools in Hyderabad, Pakistan.

**Objective:**

To determine the impact of the intervention on school-based peer violence (victimization and perpetration) and depression among school children.

**Methods:**

40 single-sex public schools were randomized into two study arms (20 per arm 10 of each sex). A total of 1752 grade 6 students (929 from intervention and 823 from control schools) were enrolled in the trial. The two-year intervention was a biweekly structured game led by a coach followed by critical reflection and discussion for 30 minutes. Primary outcomes (exposure to peer violence exhibited through victimization and perpetration and depression) were evaluated using generalized linear-mixed models.

**Results:**

Of the enrolled children (N = 1752) 91% provided data for analysis. There were significant decreases in self-reported peer violence victimization, perpetration and depression. For peer violence victimization, the reductions in the intervention and control arms were: 33.3% versus 27.8% for boys and 58.5% versus 21.3% for girls. For peer violence perpetration, the reductions were: 25.3% versus 11.1% for boys and 55.6% versus 27.6% for girls in the intervention and control arms, respectively. There were significant drops in mean depression scores (boys 7.2% versus 4.8% intervention and control and girls 9.5% versus 5.6% intervention and control).

**Conclusion:**

A well-designed and implemented play-based life-skills intervention delivered in public schools in Pakistan is able to effect a significant reduction in peer violence.

## Background

Peer violence is a problem in schools worldwide and is associated with poor school performance [1], absenteeism [2], and school dropout [3,4], as well as aggression in later life [5]. A recent systematic review suggests strong causal relationships between victimization and mental health problems such as depression, anxiety, poor general health, and suicidal ideation [6]. Peer violence is defined as a repeated experience of any act of physical, verbal, or psychological abuse of victims by peer perpetrators (i.e. fellow students, youth in the community or peer relatives at home) with the intention of causing harm [7,8]. Peer violence extends beyond harassment and bullying. There is often overlap between ‘victims’ and ‘perpetrators’ in that many of the former also go on to perpetrate violence [9]. Victims are often chosen because they are weaker than perpetrators – smaller, younger, from a minority or less desirable group or less socially connected [7]. Much of the evidence about peer violence comes from the USA [10], where 22.7% of youth reported peer violence victimization in the National Survey of Children’s Health in 2016 [11]. Prevalence estimates of peer violence in low- and middle-income countries suggest similar or higher rates [9,12,13].

The overwhelming majority of interventions to reduce peer violence are implemented and evaluated in high-income countries [3,14–16]. A recent systematic review found only three studies which evaluated interventions in low- and middle-income countries and two of the three studies had a very high risk of bias and major weaknesses in their study design, making their findings hard to interpret. Only one, from Romania, was more robust, and it included a cognitive-behavioural component to enhance students’ social and emotional learning. However, the findings showed that intervention was not effective in preventing bullying 9 months after baseline [16].

Evaluations of interventions to reduce peer victimization have been undertaken in some high-income country settings. Only cognitive-behavioural interventions have shown promise in preventing peer violence victimization and there has been little evidence of impact in follow-up assessments. Yet most peer violence interventions have focused on perpetration rather than victimization with social, emotional and cognitive components and peer mentoring showing some reductions in perpetration. Most evaluations have considered change between pre- and post-test, however evidence of the effectiveness of these interventions over the longer term, for example, one to 3 years, has also been shown [14].

Right To Play (https://www.righttoplay.ca/en-ca/) is an international non-governmental organization (NGO) that seeks to use the transformative power of play and sports to educate and empower children to lead healthier, empowered and safer lives. The Positive Child and Youth Development Programme (also called Red Ball Child Play) was developed by a team of educationists, athletes, teacher-trainers, and psychologists. The programme draws on social, cognitive, child development and experiential learning theories, focusing on physical, cognitive, social and emotional development through sports and games [17]. It is premised on the idea that children learn through processes of exploration and reinforcement of new ideas and behaviour, and that in order to achieve enduring change, interventions must be ongoing. Gender equality is a cross-cutting theme which is intended to give girls a voice through play and discussion. Behaviour change is viewed as a complex process that is best achieved when new ideas are explored in groups using empowering, participatory methods [18]. The activities seek to develop and build essential life skills, such as confidence, communication, empathy, coping with negative emotions, resilience, cooperation, leadership, critical thinking and conflict resolution in order to reduce intolerance, gender discrimination, and peer violence. The programme has been translated and adapted for different settings. In Pakistan, where the NGO has worked since 2008, the material has been translated into the Urdu and Sindhi languages. Although tested and refined over nearly 20 years across 18 countries, the programme had not been previously evaluated for long-term impact on children.

We report the results of an evaluation which assessed the impact of Right To Play’s structured play-based intervention in single-sex, public schools in Pakistan. The objective was to determine whether this play-based life skills Programme could reduce peer violence perpetration and victimization, and depression among children in 6^th^ to 8^th^ grades, who were mostly aged 12–14 years at enrollment.

## Methods

### Study setting

We evaluated the intervention in 40 public middle schools in the Hyderabad district of Sindh province, in classes from grades 6–8 between December 2015 and January 2018. A full account of the methods [19] and analyses from baseline data [9,20–22] are published elsewhere.

### Design

We conducted a two-arm cluster-randomized controlled trial with parallel group assignment. One arm received the intervention for 2 years and the control arm, received the intervention in 2018 after data collection concluded. We encouraged participation in the control arm schools by offering school-wide incentives such as providing drinking water tank or repairing school benches.

Only single-sex government schools educating students in grades 6–8 were eligible. Schools were required to have sufficiently large spaces (either indoor or outdoor) for safe play. A further requirement for inclusion was that the schools had 35 or more students in grade 6 for whom parental consent could be sought. Using the Pakistan Government’s oﬃcial 2015 schools list as a sampling frame, we found 56 potentially eligible secondary schools in Hyderabad. We excluded 16 as they had fewer than 40 enrolled students in grade 6 or were campus schools with a single administration responsible for multiple schools in the same area. We planned to include only schools that were more than 1 km away from the nearest eligible boys or girls-only school, to avoid contamination between the study arms. Forty schools met this requirement – 20 boys-only schools and 20 girls-only schools. After stratifying by gender, each school had an equal chance of being assigned to either the intervention, or control arm. We issued consent forms to all children enrolled in grade 6. We decided to recruit grade 6 students to maximize our chances of two-year follow-up.

### Sampling

Cluster randomized controlled trial software [23] was used to determine minimum sample and cluster sizes required to show statistically significant differences. Power was set at 0.80, alpha at 0.05, the inter-cluster correlation coefficient (ICC) was 0.1 and the effect size was 0.20 [24]. In the absence of local data, the effect size was guided by the findings of an evaluation of a violence prevention intervention among school students in South Africa [25]. We determined that at least 25 students per school and 20 schools per arm would be required, giving 1000 students. We were advised that in Pakistan 30% of students drop out of school between grades 6 to 8. For caution, we assumed up to 40% attrition, and inflated the minimum cluster size to 35 students per school. This yielded a recruitment goal of 1,400 students from 40 schools.

### Ethics and data collection

Permission to undertake the study was granted by the Director of School Education-Hyderabad and Principals of the potential study schools. Parental consent forms were issued to 2,486 children of which 75% (1,858) were returned with signed parental consent. Of these, 1,767 children agreed to participate (assented), 71.1% of those initially given consent forms. If parents did not provide written consent, their children did not participate in the study. However, if they were in the intervention school they were still exposed to the intervention. A clinical psychologist was available to accept referrals for any children who had difficulties during the course of the study.

We conducted baseline interviews between November and December 2015, after which the intervention commenced. The 12-month assessment was undertaken between December 2016 and January 2017 and the 24-month assessment was undertaken between November 2017 and February 2018. We declared a participant unavailable for interview after three repeat visits at midline, and five repeat visits at end line. The primary reason for attrition was students dropping out or moving schools. We lacked resources to follow up the missing children.

### Intervention

The intervention was delivered to the children by 10 male and 10 female adult coaches employed by the Right To Play NGA. They were later supported by 120 junior leaders. Further information on training and selection is published elsewhere [26]

The intervention in Pakistan was based on 103 play-based learning activities each with a specific goal as specified in the manual. Coaches selected an activity for a session from the manual. After the game, they led a three-step discussion following the formula Reflect-Connect-Apply, which involved reflection on the activity and how it made participants feel or what had been learned from it, discussion connecting this to daily life, and application more broadly to other circumstances. Over the 2 years of this study, 120 sessions were conducted in each class, with, on average, two sessions of 35 minutes per week, resulting in 60 sessions in a year. Some of the activities were conducted more than once with the class. The fidelity of the intervention was monitored by Right To Play’s staff and the main research partner, Aga Khan University. The research team verified completion of the intended number of sessions and observed a session in each school each month to ensure compliance with the manual.

In addition, the NGO organized summer camps, and also invited parents to sports tournaments and thematic play days. Right To Play also held quarterly awareness sessions with parents on child rights, gender equality and positive discipline and provided some training sessions for teachers (about three per school) on positive child and youth development, positive disciplining, gender equity, and child protection.

### Procedures

We conducted a public randomization where a Government School Superintendent drew the names of the intervention schools. Schools and children were not blinded to the study arm.

### Instruments

All measures are described in Table 1. The primary outcomes were peer victimization and peer perpetration during the previous four weeks, and symptoms of depression in the previous two weeks with the latter assessed by using Child Depression Inventory II (CDI-2) [20,27]. The intervention was intended to impact upon multiple aspects of children’s lives. We hypothesized that a reduction in peer violence would be accompanied by improved child mental health. Neither the children nor the coaches were aware that the main focus was in measuring peer violence.

**Table 1.** Table of measures used

Scale/Assessment	Characteristics	Cronbach’s Alpha and scores
**Primary outcomes**		
Multidimensional Peer-Victimization Scale^21^ Desired change ↓	16-items with 4 subscales assessing physical and verbal victimization, social exclusion, and damage to property in the last 4 weeks. Response options: never; once; few times; many times. Typical item: ‘How often within the past 4 weeks has another child done these things to you?.tripped me to make me fall’.	Peer victimization overall = 0.873 Physical = 0.673 Verbal = 0.642 Social exclusion = 0.696 Property damage = 0.658 Range 0–48
Peer Perpetration Scale^21^ Desired change ↓	16-item measure asking about perpetration of the acts measured in the victimistaion scale with the same response options and range. Typical item: ‘How often within the past 4 weeks have you … called another child bad names’.	Peer perpetration overall =.890 Physical = 0.733 Verbal = 0.696 Social exclusion = 0.723 Property damage = 0.716
Children’s Depression Inventory 2 (CDI-2) ^23^ Desired change ↓	28-item to assess depressive symptoms in last 2 weeks. Response options: no symptom; mild; definite symptom.	Alpha = 0.725 Raw scores converted to T scores (range 40–90) with ≥65 indicative of depression
**Secondary outcomes**		
Corporal Punishment at School Desired change ↓	6-items on the frequency of punishment by a teacher in the last 4 weeks, i.e., slapped, hit or beaten, made to run, kneel or stand. Response options: never, once, 2–3, or 4+ times) Typical item: ‘How often within the past 4 weeks ….Did a teacher twist your ear?’	Alpha = 0.758 Range 0–24
Parents Fighting Desired change ↓	One item on the father fighting and two on witnessing violence against their mother. Typical item: How often within the past 4 weeks …. ‘Have you seen or heard that your father had a physical fight with another man?’	These items were not a subscale and so an alpha was not calculated.
Child Gender Attitudes Desired change ↓	13-items on agreement with gender attitudes, roles and norms, such as wives obeying husbands, husbands right to punish wives, and limits to women’s participation in social events and employment. Typical item: ‘I think the wives in the family should have a say in how money in their family is spent’	Alpha = 0.738 Range 0–39
Child physical punishment at home Desired change ↓	2-items to assess frequency (i.e., never, once, 2–3 times, 4 or more times) and severity of punishment at home in the last 4 weeks. Typical item: In the past 4 weeks have you been beaten so hard at home that you were injured?	Due to only 2-items, coefficient alpha not determined.
Early Marriage Desired change ↓	2-items ‘Have you been promised in marriage to someone?’ and ‘Has your family started other preparations for your marriage?’ with yes/no responses	Not applicable
Child school performance and absence from school Desired performance change ↑ Desired change in absence ↓	4-items on self-assessed academic performance. A typical item is: “How are you doing at school [in reading and writing/Pakistan studies/maths/science]? Response options: failing, below average, average, above average. Also the number of days absent from school in the last 4 weeks and reasons for absences	Alpha = 0.642, for the four academic performance items.
Parental literacy	2 items scored: can your [mother/father] read and write? Responses: no/she reads only/she reads and writes.	Range 0–4
Going without food	2 items scored ”In the last 4 weeks, how often do you go to [school without breakfast/sleep without dinner] because of lack of food at home?” (response options: never, sometimes, every week and all or most days).	Coded as ‘never’ = no to both items, ‘sometimes’ = sometimes to one or both, ‘often’ any response of ‘every week’ or ‘most days’

Secondary outcomes were gender attitudes, roles and norms [22], school corporal punishment [21], experience of physical punishment of at home, witnessing their father or another relative hitting their mother, witnessing their father fighting with other men, self-rated school performance, number of school days missed, and preparations for marriage (Table 1). We assessed the social and demographic characteristics of the children (see Table 1). Self-reported absences were validated against school records.

Instruments were translated into Urdu and Sindhi and self-completed, however a fieldworker read out the questions to a group of four children to facilitate comprehension. We ensured children did not copy answers and reassured them repeatedly that there were no right or wrong answers. Further information on the instruments is given elsewhere [19].

### Statistical analysis

We defined study loss to follow-up as missing at both the 12 and 24 months interviews. The final analysis excluded those lost to follow-up. Because the percentage of missing data for items was low, ranging from 0.1% to 3.6%, and mostly with just one missing item, we did not impute missing data. We examined items for randomness in missing data using Little’s covariate-dependent missingness (CDM) test. The test showed no association between missing data and baseline covariates.

For all outcomes, except for ‘days of school missed’, ‘depression’ and ‘preparation for marriage’, we derived a score by adding the items. For depression, we derived raw scores for the CDI-2 scale and then converted these to T-scores as described in the CD1-2 technical manual [27]. A student was deemed to have ‘preparation for marriage’ if they responded yes to either of the two questions. We assessed the percentage change in mean scores for the continuous outcomes, or in the proportion of learners ‘promised/prepared for marriage’ from baseline to 24 months’ post-baseline.

Cohen’s standardized difference was used to compare study outcomes and socio-demographic characteristics at baseline between the two study arms. Logistic regression tested for association between loss to follow up and study arm, socio-demographic characteristics, and baseline levels of all outcomes for both boys and girls. Generalized linear mixed effects modelling (multi-level model for change) with the Gaussian link function was used to compare mean scores at end-line for all outcomes except for the binary outcome of ‘preparation for marriage’. Full information maximum likelihood estimation was used to deal with missing data in study outcomes due to missed interviews at 12 or 24 months post-baseline. For the binary outcome, a generalized linear mixed effects model with a logit link function was used to compare the effect of the intervention between the two study arms. The fixed effects terms included study arm, gender and data collection wave, an interaction term for study arm and data collection wave, and an interaction term for study arm and gender. School was added as the random effects term. The Kenward-Roger method was used to calculate denominator degrees of freedom due to the small number of clusters in each study arm for boys and girls [28]. The effect of the intervention was assessed at 24 months using linear combinations of the fixed effect terms in the model (study arm, gender and data collection wave). We compared boys from the control and intervention arm, and girls from control and intervention arm. Residual plots were computed for each continuous outcome to check for distribution assumptions. For each outcome, we assessed standardized residuals from the model without the random component as well as from the model with random components.

As a sensitivity analysis, we used generalized linear mixed effects modelling to perform a cluster-level analysis where all outcomes were aggregated at school level (mean scores for continuous outcomes and proportions for binary outcomes). The cluster-level model was adjusted for baseline outcomes aggregated at cluster level, and we used the Kenward-Roger method. Analyses were conducted in Stata 14 with statistical significance set at 5%.

## Results

### Trial profile

**Figure 1.**
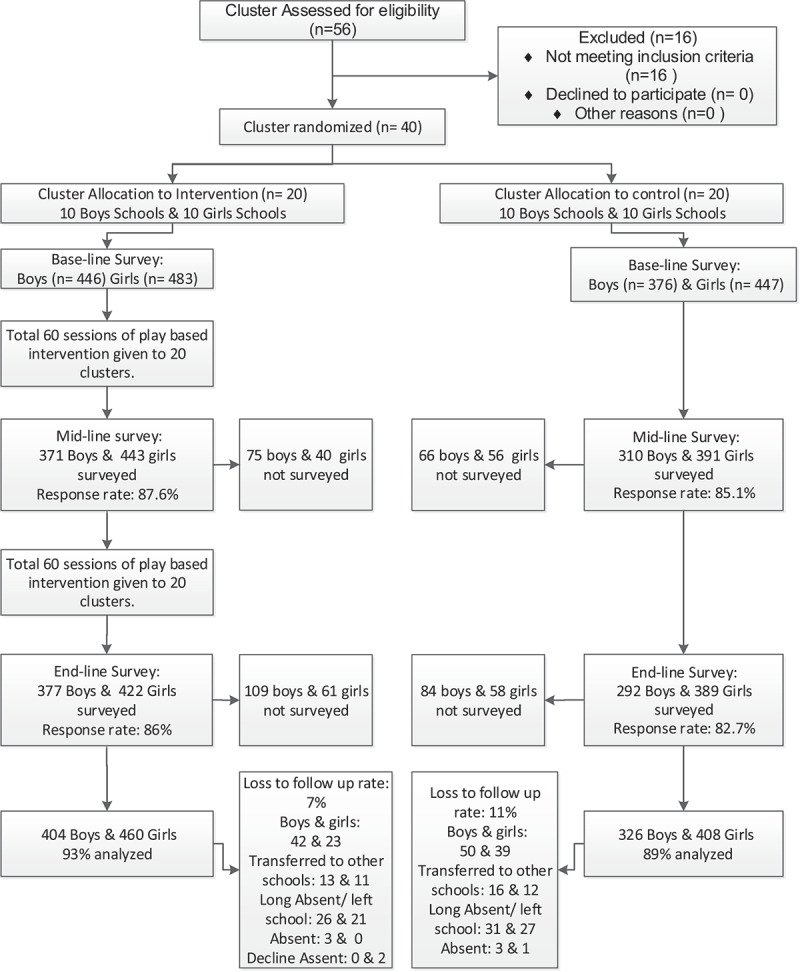
Trial profile

The trial profile is shown in Figure 1. A total of 446 boys and 483 girls from the intervention arm and 376 boys and 447 girls from the control arm were enrolled in the trial. The 12 months follow-up rate was 86.5% (1515/1752) and at 24 months, it was 84.5% (1480/1752). Of the 1752 learners enrolled into the study, 1397(79.7%) were interviewed at all the three data collection points, 118 (6.7%) were available at baseline and 12 months only, 83 (4.7%) were available at baseline and the 24 months only. No schools (clusters) were lost to follow up. Loss to follow up of participants is shown in Figure 1. Reasons for loss were children having transferred to other schools, long absence from schools, and for boys, having left schools for employment, and for girls, for household chores. Two girls had married. There were no reports of serious adverse events.

### Background characteristics

Table 2 shows the participants’ baseline characteristics. Most participants were aged 12–14 years and many of the participants were not food secure, sometimes or often going without food. Between 20% and 25% had repeated an academic grade. Many were exposed to violence at home and in the community including witnessing their father fighting with another man and their mother being beaten by their father or another relative in the past month.

**Table 2.** Baseline social and demographic characteristics of participants from control and intervention schools and study outcomes

	Boys						Girls					
		Control		Intervention				Control		Intervention		
	N	n/mean	%/sd	n/mean	%/sd	Std Diff.	N	n/mean	%/sd	n/mean	%/sd	Std Diff.
**Age group** ≤10 yrs	51	23	6.1	28	6.3	0.11	79	34	7.6	45	9.3	0.18
11 yrs	150	63	16.8	87	19.5		187	85	19.0	102	21.1	
12 yrs	258	128	34.1	130	29.1		307	139	31.1	168	34.8	
13 yrs	175	77	20.5	98	22.0		196	97	21.7	99	20.5	
≥ 14 yrs	187	84	22.4	103	23.1		161	92	20.6	69	14.3	
**Without food** Never	536	242	64.4	294	65.9	0.06	685	328	73.4	357	73.9	0.02
Sometimes	132	59	15.7	73	16.4		120	59	13.2	61	12.6	
Often	154	75	19.9	79	17.7		125	60	13.4	65	13.5	
**Number of rooms in household**												
≤2 rooms	368	187	49.9	181	40.9	0.30	441	226	50.67	215	44.5	0.13
3–5 rooms	339	157	41.8	182	41.2		365	161	36.1	204	42.2	
≥ 6 rooms	110	31	8.3	79	17.9		123	59	13.2	64	13.3	
**Ever repeated a grade**	208	101	26.9	107	24.2	0.06	196	91	20.4	105	21.9	0.04
**SES score (mean)**	822	4.3	0.66	4.4	0.7	0.02	930	4.4	0.6	4.2	0.5	0.24
**Number of people in household (mean)**	822	9.2	6	10	5.9	0.13	929	10	5.9	9.6	5.2	0.12
**Number of siblings (mean)**	822	4.7	2.3	5	2.4	0.11	931	4.9	2.3	4.8	2.2	0.06
**Parent’s literacy score (mean)**	822	2.5	1.4	2.7	1.4	0.11	931	2.5	1.4	2.5	1.4	0.001
**Witnessing father fighting with other men**	211	98	26.1	113	25.5	0.01	166	75	16.8	91	18.9	0.05
**Witnessing mother beaten by father or relatives**	106	50	13.3	56	12.58	0.07	77	32	7.2	45	9.3	0.08
**Peer violence victimization score**	822	12.6	9.7	12.3	8.2	0.04	930	6.1	6.7	8.2	7.9	0.24
**Peer violence perpetration score**	822	7.2	7.7	7.5	7.4	0.03	930	2.9	4.4	3.6	5.1	0.15
**Depression score**	822	56.4	9.9	56.8	9.6	0.02	930	53.6	8.6	55.5	9.7	0.18
**Gender attitudes score**	822	18.6	5.3	18.6	5.5	0.02	930	16.4	4.8	16.7	5.4	0.05
**School performance**	821	9.4	1.8	9.3	1.7	0.03	929	9.7	1.8	9.5	1.6	0.06
**Corporal punishment at school score**	822	5.0	3.8	4.7	3.5	0.11	930	1.2	1.7	1.7	2.1	0.27
**Physical punishment at home score**	822	1.1	1.2	1.1	1.3	0.01	930	0.54	0.91	0.56	0.84	0.02
**Number of days of school missed**	813	4.0	4.0	4.0	4.1	0.01	927	3.0	2.7	3.2	2.9	0.03

Std Diff = standardized difference (used to compare characteristics between treatment arms). Std Diff >0.20 indicates some significant differences between arms.

There were no significant differences in socio-demographic characteristics between intervention and control arms for both boys and girls and no significant differences at baseline in any of the study outcomes among boys. However, girls in the intervention arm had slightly higher mean scores for peer violence victimization, depression or corporal punishment than control arm girls (effect size≥0.2).

### Primary outcomes

Table 3 shows the results of analysis of the effect of the intervention on the primary outcomes peer violence victimization, peer violence perpetration and depression for boys and girls at 24 months. After adjusting for the baseline level of each variable, there were statistically significant differences in mean scores at end-line between control and intervention arms. For boys, the percentage reduction in the mean peer-victimization score was 27.8% in the control versus 33.3% in the intervention arm. For girls, the percentage reduction in this measure was 21.3% in the control versus 58.5% in the intervention arm. For boys, the percentage reduction in the mean peer perpetration score was 11.1% in the control versus 25.3% in the intervention arm. For girls, the percentage reduction in this was 27.6% in the control versus 55.6% in the intervention arm. The mean depression score in boys dropped by 4.8% in the control versus 7.2% in the intervention arm and in girls it dropped by 5.6% in the control versus 9.5% in the intervention arm.

**Table 3.** Results of analysis of the effects of the intervention on primary outcomes for boys and girls

		Mean scores over time				Model results			
		Baseline	Midline	Endline	% change between baseline and endline	EMD	LCL	UCL	P value
**Boys**									
Peer victimization score	Control	12.6	9.8	9.1	27.8				
	Intervention	12.3	11.7	8.2	33.3	−1.57	−2.56	−0.58	0.002
Peer perpetration score	Control	7.2	6.6	6.4	11.1				
	Intervention	7.5	6.9	5.6	25.3	−1.18	−1.91	−0.45	0.002
Depression scores	Control	56.4	54.9	53.7	4.8				
	Intervention	56.8	56.0	52.7	7.2	−1.91	−2.98	−0.85	<0.001
**Girls**									
Peer victimization score	Control	6.1	5.7	4.8	21.3				
	Intervention	8.2	7.3	3.4	58.5	−1.98	−2.95	−1.02	<0.001
Peer perpetration score	Control	2.9	2.9	2.1	27.6				
	Intervention	3.6	3.8	1.6	55.6	−0.79	−1.5	−0.08	0.029
Depression scores	Control	53.6	51.8	50.8	5.2				
	Intervention	55.5	55.1	50.2	9.5	−1.11	−2.13	−0.08	0.034

### Secondary outcomes

Table 4 shows the changes observed in the secondary outcomes. Gender attitudes changed significantly for boys and girls, becoming less patriarchal in the intervention arm than the control arm, although the reduction in scores was greater for girls than boys. Corporal punishment was reported significantly less often during the last four weeks, by both boys and girls in the intervention arm compared to the control arm, with the reduction greater for girls. Physical punishment at home during the last four weeks was reported significantly less often in the intervention arm, with a greater reduction for girls than boys. The difference in the proportion promised in marriage for boys and girls showed possible evidence in change in the desired direction, but was not statistically significant.

**Table 4.** Results of analysis of the effects of the intervention on secondary outcomes for boys and girls

		Mean scores over time				Model results			
**Boys**		Baseline	Midline	Endline	% change baseline to endline	EMD	LCL	UCL	P value
Gender attitudes scores (low = good)	Control	18.6	16.2	16.3	12.4				
	Intervention	18.6	17.1	16.0	14.0	−0.65	−1.27	−0.04	0.037
School performance score	Control	9.4	9.1	9.2	−2.1				
	Intervention	9.3	9.1	9.3	0.0	0.12	−0.05	0.29	0.178
Corporal punishment experience at school	Control	5	4.4	3.6	28.0				
	Intervention	4.7	4	2.6	44.7	−0.80	−1.15	−0.45	<0.001
Physical punishment experience at home	Control	1.1	0.67	0.52	52.7				
	Intervention	1.1	0.66	0.42	61.8	−0.14	−0.24	−0.04	0.005
Number of days missed	Control	4	4.4	6.8	−70.0				
	Intervention	4	4.2	6.2	−55.0	−0.28	−0.97	0.42	0.440
Promised in marriage (%)	Control	8.9	7.1	8.9	0.0				
	Intervention	7.8	7.6	6.4	17.9	0.69*	0.38	1.25	0.226
**Girls**									
Gender attitudes scores (low = good)	Control	16.4	14.5	14.1	14.0				
	Intervention	16.7	14.1	13.7	18.0	−1.32	−1.92	−0.73	<0.001
School performance score	Control	9.7	9.8	9.9	2.1				
	Intervention	9.5	9.4	9.7	2.1	−0.07	−0.24	0.09	0.372
Corporal punishment experience at school	Control	1.2	1.2	0.82	31.7				
	Intervention	1.7	1.5	0.58	65.9	−0.47	−1.14	−0.46	<0.001
Physical punishment experience at home	Control	0.54	0.43	0.27	50.0				
	Intervention	0.56	0.42	0.13	76.8	−0.14	−0.24	−0.05	0.003
Number of days missed	Control	3.0	3.3	4.1	36.7				
	Intervention	3.2	3.5	4.6	43.8	0.13	−0.55	0.81	0.702
Promised in marriage (%)	Control	3.2	5.6	4.9	53.1				
	Intervention	5.9	4.3	4.5	23.7	0.63*	0.34	1.16	0.131

EMD: Estimated mean difference in scores between intervention and control groups.

*Adjusted odds ratio.

Two of the secondary outcomes did not show evidence of desired change: the self-assessed school performance score and the number of days of school missed in the previous four weeks. The latter was higher at 24 months for both boys and girls than at baseline. For boys, it was 77% higher in the control arm versus 55% higher in the intervention arm, and for girls, it was 36.7% in the control arm and 43.8% higher for girls in the intervention arm. These differences were not statistically significant.

### Exploratory outcomes

Table 5 shows an analysis of three exploratory outcomes: experiences of hunger, witnessing of fighting between the child’s father and another man, and witnessing the child’s mother being beaten at home. For both boys and girls, these outcomes were reported significantly less often reported among children in the intervention arm compared to the control arm at end line. The results of the sensitivity analysis performed at cluster level were consistent with the individual-level analysis above.

**Table 5.** Effect of intervention on other exploratory outcomes for boys and girls

Outcome		All		Control		Intervention			Control vs Intervention at 24 m
Boys		N	%	n	%	n	%	OR	P value
Without food often	baseline	252	34.5	112	34.4	140	34.7		
	12 m	135	19.8	64	20.7	71	19.1		
	24 m	69	10.3	40	13.7	29	7.7	0.36(0.24–0.55)	<.001
Witnessing mother beaten by father or relatives	baseline	90	12.3	41	12.6	49	12.1		
	12 m	46	6.8	21	6.8	25	6.7		
	24 m	33	4.9	17	5.8	16	4.2	0.53(0.28–0.99)	.047
Witnessing father fighting with other men	baseline	190	26.1	85	26.1	105	26.1		
	12 m	128	18.8	50	16.1	78	21.0		
	24 m	67	10.0	35	12.0	32	8.5	0.59(0.39–0.90)	.015
**Girls**									
Without food often	baseline	226	26.0	104	25.4	122	26.5		
	12 m	124	14.9	57	14.5	67	15.1		
	24 m	93	11.5	63	16.2	30	7.1	0.34(0.23–0.53)	<.001
Witnessing mother beaten by father or relatives	baseline	71	8.2	27	6.6	44	9.6		
	12 m	48	5.8	23	5.9	25	5.6		
	24 m	30	3.7	18	4.6	12	2.9	0.47(0.24–0.90)	0.022
Witnessing father fighting with other men	baseline	156	18.0	67	16.4	89	19.4		
	12 m	134	16.1	66	16.8	68	15.4		
	24 m	85	10.5	52	13.3	33	7.8	0.44(0.29–0.67)	<.001

## Discussion

We have shown that Right to Play’s Positive Child and Youth Development Programme in Pakistan, delivered under our study conditions, enabled a significant reduction in peer violence victimization, and perpetration and depression among boys and girls in the intervention schools compared to the control schools. Girls reported a greater decline in violence outcomes than boys. The programme showed positive impact on gender attitudes, corporal punishment, and child physical punishment at home, again with greater changes reported by girls. The reduction in depression adds confidence to the interpretation of these findings, as depression is a well-established consequence of peer violence and the complexity of the CDI-II measure enhances response validity.

This research has advanced the small body of evidence on the prevention of peer violence victimization and perpetration in low- and middle-income countries in general, and Pakistan in particular. It is the first evaluation of an intervention here to show positive impact. Globally, this is the first evaluation of the impact of a play-based life-skills intervention on peer violence among school children. The findings show that the intervention is effective in preventing violence victimization and perpetration amongst girls as well as boys in and out of school settings, as well as some other forms of out of school violence. This intervention is particularly important because evidence shows that school violence has far-reaching effects and that different forms of violence are interconnected [29]. For example, peer violence perpetrators are more likely to engage in dating and partner violence [30]. Using schools as an entry point for reducing violence overall is particularly important in a country like Pakistan which has relatively high levels of culturally sanctioned violence and where oppression of women and girls is widespread.

We found greater violence reduction reported by girls than boys, and this may be because teenage girls are more mature than boys and so may benefit more from an intervention that requires discussion and processing of information. It is also possible that boys’ violence is harder to prevent. Peer violence in Pakistan and many other countries is more socially acceptable behaviour for boys than girls, and consequently, there is a higher prevalence among boys, and this may explain why it is harder to change their behaviour [31].

We did not show an impact on self-reported school performance measures nor on child-reported days missed from school. We recognize that the main drivers of days missed (child labour and help with domestic chores) were not changed through this intervention and the measures were self-reported. We were pleased to see that the children did not give socially desirable answers to these questions, and this further enhances our trust in the other reported outcomes. The changes in peer violence and depression were recorded in both intervention and control arms, as is common in randomized-controlled trials. This may be associated with age-related changes in peer violence and associated depression, or be an artefact of the repeated measurements in the trial [32].

### Strengths and limitations

The study strengths included its large sample, drawn from poorer schools in a major city in Pakistan, and the fact no schools dropped out. Our results may be generalizable to similar settings in Pakistan. The likelihood of replicability of the results in another setting is enhanced by the long-established approach to work to Right To Play. The study was well powered, even after taking into account changes in the control arm, retention was high and there was little missing data. The evaluation was conducted by an independent research team and the involvement of Right To Play staff was limited to delivering the intervention. The coaches were not informed of the primary outcome measures. A key limitation was that the outcome measures were self-reported and we had to assume that the ‘last four weeks’ was typical, but reporting biases should have been similar between the two study arms. Our confidence in our results was enhanced by the concurrent findings of reduction in depression, and the fact that we did not see positive changes in all of our measures.

The evaluation had features of both an effectiveness and efficacy trial. We did not consider individual-level dose in the analysis and did not influence intervention attendance. In this evaluation the coaches were trained for a little longer than was usual due to a research-related delays in starting the intervention, and the research team’s oversight of the sessions delivered may have strengthened adherence to the manual. Right To Play has now adopted these changes into the normal practice.

## Conclusion

We found the play-based life-skills intervention of Right To Play, when delivered over 2 years to children in grades 6–8 in Hyderabad, Pakistan, reduced peer violence in school and family violence in the home. Play-based life-skills interventions thus constitute a new dimension in the repertoire of effective approaches to prevent peer violence and should be evaluated in other settings. Further research is needed to determine whether the impact is sustained beyond the 2 years of a study and whether positive impact persists if the intervention is taken to scale. This study increases understanding of the types of interventions that are effective in reducing peer violence in low- and middle-income countries. This study has broadened our understanding of the types of interventions that are effective in reducing peer violence in low- and middle-income countries.

## Paper context

Systematic reviews have observed that there has been little research evaluating interventions to prevent peer violence in low- and middle-income settings. This paper describes an evaluation of an intervention in Pakistan which was found to be effective in preventing peer violence perpetration and victimization. This is the first time a rigorous evaluation of a structured play-based life-skills programme has been conducted and shown impact on peer violence prevention. It is the first time any peer violence prevention intervention in a low- and middle-income country setting has been evaluated and shown to be impactful. Pakistan is a country in which violence is highly prevalent and so it is important that interventions are shown to work in this context. The study represents an important advance in peer violence prevention globally. Research is encouraged in other regions.
